# A phase 1 dose-escalation study of the oral histone deacetylase inhibitor abexinostat in combination with standard hypofractionated radiotherapy in advanced solid tumors

**DOI:** 10.18632/oncotarget.14147

**Published:** 2016-12-24

**Authors:** Eric Deutsch, Elizabeth Cohen-Jonathan Moyal, Vanesa Gregorc, Paolo Andrea Zucali, Jean Menard, Jean-Charles Soria, Ioana Kloos, Jeff Hsu, Ying Luan, Emily Liu, Remus Vezan, Thorsten Graef, Sofia Rivera

**Affiliations:** ^1^ Department of Radiation Oncology, Gustave-Roussy Cancer Campus, Villejuif, France; ^2^ INSERM 1030 Molecular Radiotherapy, Villejuif, France; ^3^ Faculté de Médecine, Université Paris-Sud, Université Paris-Saclay, Le Kremlin-Bicêtre, France; ^4^ Department of Radiation Oncology, Institut Claudius Regaud, Toulouse, France; ^5^ Department of Medical Oncology, Istituto di Ricovero e Cura a Carattere Scientifico, Ospedale San Raffaele, Milan, Italy; ^6^ Department of Medical Oncology and Haematology, Humanitas Cancer Center, IRCCS, Rozzano, Italy; ^7^ Department of Radiation Oncology, Hopital Saint-Louis, Paris, France; ^8^ DITEP (Département d’Innovations Thérapeutiques et Essais Précoces), Gustave Roussy Cancer Campus, Villejuif, France; ^9^ Institut de Recherches Internationales Servier, Clinical Pharmacokinetics, Suresnes, France; ^10^ Pharmacyclics LLC, an AbbVie Company, Sunnyvale, CA, USA

**Keywords:** abexinostat, histone deacetylase inhibitor, radiotherapy, solid tumors, brain lesions

## Abstract

Current treatments for advanced solid tumors tend to be only palliative. Although radiotherapy is administered with a curative intent, radioresistance and dose-limiting toxicities pose limitations to treatment. Abexinostat, an oral pan-histone deacetylase inhibitor, demonstrated enhanced sensitivity to radiation in various solid tumor cell lines. We conducted an exploratory, phase 1, dose-escalation study of abexinostat in combination with standard hypofractionated radiotherapy in patients with advanced solid tumors treated in a palliative setting. Among 58 treated patients, the median age was 61.5 years (range, 20-82); 47% of the patients had M1 stage disease, and 95% had received previous chemotherapy alone or chemotherapy in combination with surgery and/or radiotherapy. The recommended phase 2 dose was determined to be 90 mg/m^2^ (140 mg). Of the 51 patients evaluable for response, best overall response was 8% (1 complete response [CR], 3 partial responses [PRs]), and best loco-regional response was 12% (1 CR and 5 PRs) at a median follow-up of 16 weeks. Of note, patients with target or non-target brain lesions showed encouraging responses, with 1 patient achieving a best loco-regional response of CR. Treatment-emergent grade ≥3 adverse events (AEs) were few, with most common being thrombocytopenia (17%), lymphopenia (12%), and hypokalemia (7%). Six patients (10%) discontinued treatment due to AEs. No grade ≥3 prolongation of the QTc interval was observed, with no treatment discontinuations due to this AE. Oral abexinostat combined with radiotherapy was well tolerated in patients with advanced solid tumors. The combination may have potential for treatment of patients with brain lesions.

## INTRODUCTION

Epigenetic changes such as DNA methylation and post-translational histone acetylation play a critical role in the development of cancer due to their ability to alter the accessibility of transcription factors to DNA and chromatin structure [1]. Histone deacetylases (HDACs) enzymatically remove acetyl groups from histones and thus serve as key regulators of gene expression. Tumorigenesis is linked to aberrant activity of HDACs such as deacetylation of the tumor suppressor gene *p53*, leading to its decreased transcription [2], and HDAC-mediated upregulation of oncogenes such as *BCL2* [3]. The US Food and Drug Administration has approved the HDAC inhibitors (HDACIs) vorinostat and romidepsin for the treatment of cutaneous T-cell lymphoma, and romidepsin and belinostat are approved for the treatment of peripheral T-cell lymphoma. Panobinostat in combination with bortezomib and dexamethasone has been approved for the treatment of multiple myeloma. These agents have also been evaluated as single agents in a variety of solid tumors but have shown limited or no activity [4–11]. Due to the broad specificity and moderate activity of the existing HDACIs observed in these studies, a combinatorial approach has been suggested.

Abexinostat is an oral, broad-spectrum, phenyl hydroxamic acid-based pan-HDAC inhibitor that has demonstrated antitumor activity as a single agent in neuroblastoma cell lines [12], as well as in combination with bortezomib in mouse xenograft models [12]. Additionally, abexinostat as a single agent and in combination with chemotherapy has demonstrated significant antiproliferative activity in human soft tissue sarcoma models [13]. Abexinostat-induced apoptosis is known to occur through caspase-8 and the Fas-associated death domain and is associated with a prominent increase in reactive oxygen species [14].

In recent years, HDACIs are emerging as promising radiosensitizing agents that play a critical role in cellular processes, such as cell growth and differentiation, apoptosis, and DNA repair [15]. Pre-treatment with abexinostat enhanced sensitivity to radiation in prostate, colon, lung, and cervical tumor cell lines [16, 17], supporting the clinical role for HDACIs in radiosensitization. HDAC inhibition by abexinostat led to a decrease in the number of cells able to form colonies after irradiation compared with radiation alone. Pre-treatment of colon tumor cells with abexinostat before irradiation induced a strong inhibition of RAD51-containing subnuclear repair foci formation, suggesting that abexinostat may act, in part, by inhibiting DNA repair [17]. Based on the pre-clinical studies showing activity of abexinostat in solid tumor models and enhanced radiosensitization by abexinostat in solid tumor cell lines, an exploratory phase 1, dose escalation study of abexinostat in combination with standard hypofractionated radiotherapy was conducted in patients with advanced solid tumors treated in a palliative setting.

## RESULTS

### Patients

A total of 62 patients were enrolled in the study between September 2, 2010, and March 25, 2015. Fifty-eight patients received at least 1 dose of the study treatment, of whom 33 patients were treated in schedule 1 and 25 patients in schedule 2. Four patients were withdrawn from the study (3 due to AEs and 1 due to protocol deviation). The number of patients enrolled by schedule and group (supra- and subdiaphragmatic) is shown in Supplementary Table S1. Baseline characteristics of all treated patients are summarized in Table 1. The median age was 61.5 years (range, 20-82), with 38% of patients aged 65 years. The most common primary diagnoses were breast cancer (36%), followed by lung cancer (24%), colorectal cancer (9%), and neuroendocrine cancer (9%); 33% of the patients were staged T2 at baseline, 21% T3, and 17% T4. Overall, 47% of the patients had M1 stage disease and 43% M0. Of the 58 treated patients, 95% had received previous chemotherapy alone or chemotherapy in combination with surgery and/or radiotherapy. Overall, 33% of patients received ≥4 lines of chemotherapy. The most prior common chemotherapy regimens included fluorouracil (40%), cisplatin (33%), cyclophosphamide (29%), docetaxel (28%), and epirubicin (22%). No patient had received surgery or radiotherapy alone, and only 1 patient (2%) had received a combination of surgery and radiotherapy. Two patients (3%) had received no previous therapy for disease control.

**Table 1:** Baseline characteristics by schedule 1 and 2

Characteristic	Schedule 1 (n=33)	Schedule 2 (n=25)	Schedule 1 + Schedule 2 (N=58)
Median age (range), years	61 (20-76)	61 (37-82)	61.5 (20-82)
>65 years, n (%)	13 (39%)	9 (36%)	22 (38%)
Males, n (%)	13 (39%)	8 (32%)	21 (36%)
Eastern Cooperative Oncology Group performance status, n (%)			
Normal activity without restriction	17 (52%)	6 (24%)	23 (40%)
Ambulatory but restricted in physical activity	15 (46%)	14 (56%)	29 (50%)
Ambulatory but unable to carry out any work	1 (3%)	5 (20%)	6 (10%)
Limited self-care, 50% in bed	0	0	0
Completely disabled, self-care limited	0	0	0
Types of prior therapies, n (%)			
Antineoplastic agents	30 (91%)	22 (88%)	52 (90%)
Endocrine therapy	7 (21%)	7 (28%)	14 (24%)
All other therapeutic products	9 (27%)	4 (16%)	13 (22%)
Drugs for treatment of bone diseases	0 (0%)	3 (12%)	3 (5%)
Pituitary and hypothalamic hormones and analogues	1 (3%)	1 (4%)	2 (3%)
Antiemetics and antinauseants	1 (3%)	0 (0%)	1 (2%)
Diagnostic radiopharmaceuticals	1 (3%)	0 (0%)	1 (2%)
Immunostimulants	1 (3%)	0 (0%)	1 (2%)
Sex hormones and modulators of the genital system	1 (3%)	0 (0%)	1 (2%)
Number of prior chemotherapy lines, n (%)			
1	6 (18%)	3 (12%)	9 (16%)
2	7 (21%)	7 (28%)	14 (24%)
3	9 (27%)	4 (16%)	13 (22%)
≥4	11 (33%)	8 (32%)	19 (33%)
Median number of prior therapy lines (range)	3 (1-10)	3 (1-8)	3 (1-10)
Primary diagnosis, n (%)			
Breast cancer	10 (30%)	11 (44%)	21 (36%)
Lung cancer	7 (21%)	7 (28%)	14 (24%)
Colorectal cancer	5 (15)	0 (0%)	5 (9%)
Neuroendocrine cancer	3 (9%)	2 (8%)	5 (9%)
Malignant melanoma	3 (9%)	0 (0%)	3 (5%)
Other cancers	5 (15%)	5 (20%)	10 (16%)

In the supradiaphragmatic group, the common sites of irradiation included lung/mediastinum (n=13 patients) and cerebral (n=10), followed by bone lesions (n=7 including 2 scapular, 2 vertebral, 2 sternum, and 1 rib lesion), other thoracic soft tissue (n=4), and cervical (n=3). In the subdiaphragmatic group, the common sites of irradiation were bone lesions (n=12 including 6 vertebral, 2 femoral, and 4 iliac lesions), followed by other soft tissue (n=4 including 1 abdomen, 1 hip, 1 suprarenal, and 1 thigh lesion), pelvis (n=4), and finally organ lesions (n=2) and sites of adenopathy (n=3).

At a median follow-up of 16 weeks (range, 0.6, 52.6), 83% of all treated patients completed the study. Of the 58 treated patients, 10 patients (17%) discontinued treatment. The primary reason for discontinuation was AEs in 6 patients (10%), progressive disease in 2 patients (3%), protocol deviation in 1 patient (2%), and nonmedical reason in 1 patient (2%). No patient was lost to follow-up (Table 2).

**Table 2:** Patient disposition by schedule

All Treated, n (%)	Schedule 1 (n=33)	Schedule 2 (n=25)	Schedule 1 + Schedule 2 (N=58)
Completed	28 (85%)	20 (80%)	48 (83%)
Withdrawn due to	5 (15%)	5 (20%)	10 (17%)
Adverse event	4 (12%)	2 (8%)	6 (10%)
Protocol deviation	1 (3%)	1 (4%)	2 (3%)
Progressive disease	0 (0%)	1 (4%)	1 (2%)
Nonmedical reason	0 (0%)	1 (4%)	1 (2%)
Lost to follow-up	0 (0%)	0 (0%)	0 (0%)

### Efficacy

Of the 51 patients evaluable for response, best overall response was 8% (1 CR, 3 PRs), and best loco-regional response was 12% (1 CR and 5 PRs) at a median follow-up of 16 weeks (Table 3). The best overall and loco-regional response by supra- and subdiaphragmatic group is shown in Supplementary Table S2.

**Table 3:** Best overall and loco-regional response by schedule

Best ORR, n (%)	Schedule 1 (n=30)	Schedule 2 (n=21)	Schedule 1 + Schedule 2 (N=51)
CR	0 (0.0%)	1 (5%)	1 (2%)
PR	1 (3%)	2 (10%)	3 (6%)
SD	17 (57%)	10 (48%)	27 (53%)
PD	10 (33%)	8 (38%)	18 (35%)
Non-CR/Non-PD	2 (7%)	0 (0%)	2 (4%)
Objective response rate	1 (3%)	3 (14%)	4 (8%)
95% CI	0.1-17.2	3.0-36.3	2.2-18.9
**Best Loco-regional Response Rate, n (%)**	**Schedule 1(n=30)**	**Schedule 2(n=21)**	**Schedule 1 + Schedule 2(N=51)**
CR	0 (0%)	1 (5%)	1 (2%)
PR	1 (3%)	4 (19%)	5 (10%)
SD	21 (70%)	11 (52%)	32 (63%)
PD	4 (13%)	4 (19%)	8 (16%)
Non-CR/Non-PD	1 (3%)	1 (5%)	2 (4%)
Objective response rate	1 (3%)	5 (24%)	6 (12%)
95% CI	0.1-17.2	8.2-47.2	4.4-23.9

Ten patients had brain lesions as target or non-target lesions, with the most common primary tumor being breast adenocarcinoma (n=6), followed by 1 case each of adenocarcinoma of the colon, adenocarcinoma of the esophagus, adenocarcinoma of the lung, and melanoma. Loco-regional efficacy assessment was performed in 7 patients. Of 4 patients with brain lesions as target lesions, 1 patient achieved a loco-regional best response of PR, and 3 achieved stable disease (loco-regional response rate 25%). Of 6 patients with brain lesions recorded as non-target lesions, the loco-regional best responses were 1 CR, 4 non-CR/non-PD, and 1 PD (loco-regional response rate 17%). The primary tumor histology for the patient achieving a CR was lung adenocarcinoma. Three patients did not complete the tumor evaluation visit at week 6 as per protocol requirement (they did complete the follow-up visit) and were therefore considered non-evaluable for final efficacy assessment.

### Safety

#### MTDs and DLTs for schedule 1 and 2

Abexinostat was administered BID from day 1 to 5 and during radiotherapy from day 8 to 12 and 15 to 19. This schedule of 5 days on/2 days off (schedule 1) chosen for this study was used with a starting daily dose of 15 mg/m^2^ BID.

The schedule of 4 days on/3 days off (schedule 2) was based on tolerability of abexinostat given as stand-alone therapy observed in the clinical study CL1-78454-002 [18], a study performed during the same timeframe. This schedule is associated with the smallest platelet decrease [18], and dose levels 75 mg/m^2^ BID, 90 mg/m^2^ BID, and 105 mg/m^2^ BID. Changes in the abexinostat administration schedule were made to increase the hematological therapeutic window of abexinostat.

Twenty-seven patients were evaluated for DLT in schedule 1. MTD1 for schedule 1 was reached at 60 mg/m^2^ in the supradiaphragmatic group (2 DLTs for 6 evaluable patients), after which dose escalation was initiated from MTD1 (60 mg/m^2^ BID) with schedule 2 of administration (i.e., 4 days on/3 days off per week over 2 weeks). MTD1 of schedule 1 was not reached at 60 mg/m^2^ BID in the subdiaphragmatic group, after which dose escalation started from the next dose level (75 mg/m^2^ BID) with schedule 2.

Twenty-two patients were evaluated for DLT in schedule 2. MTD2 of schedule 2 was reached at 105 mg/m^2^ (160 mg) for the subdiaphragmatic group. Four patients treated at the dose level of 105 mg/m^2^ (160 mg) across supra/subdiaphragmatic groups were evaluable for assessment of DLTs. Numbers of patients and DLTs at each dose level within the supra- and subdiaphragmatic groups are given in Supplementary Table S3.

At the highest dose level of 105 mg/m^2^ (160 mg), 3 out of 4 evaluable patients had a DLT (2 in subdiaphragmatic group and 1 in supradiaphragmatic group) of hematologic or gastrointestinal origin (2 cases of grade 4 thrombocytopenia and 1 case of grade 3 diarrhea). No clinically meaningful differences were observed between the supra- and subdiaphragmatic groups in terms of frequencies of DLTs. Based on these DLT observations, the study sponsor and study investigators determined that MTD2 was reached at 105 mg/m^2^ (160 mg), and the recommended phase 2 dose was therefore considered 90 mg/m^2^ (140 mg) for both subdiaphragmatic and supradiaphragmatic groups.

Overall, 98% of all treated patients experienced 1 or more AE. Grade ≥3 AEs occurred in 55% of patients. Grade ≥3 hematologic AEs occurring in more than 1 patient included thrombocytopenia (n=10, 17%), lymphopenia (n=7, 12%), and neutropenia (n=2, 3%) (Table 4). Grade ≥3 nonhematologic AEs occurring in more than 1 patient included hypokalemia (n=4, 7%), asthenia (n=3, 5%), diarrhea (n=2, 3%), gamma-glutamyltransferase increased (n=2, 3%), and decreased appetite (n=2, 3%) (Table 4). No substantial differences were observed in the frequencies of AEs between the supra- and subdiaphragmatic groups. Seventeen patients (29%) experienced an SAE, which were grade ≥3 in 22% of patients. Treatment-related SAEs occurred in 12% of patients (9% grade ≥3). Malignant neoplasm progression leading to death was reported in 3 patients (5%).

**Table 4:** Overview of common AEs (>10%) and grade ≥3 AEs in >1 patient

Hematologic AEs, n (%)	AE (>10%)	Any grade	Grade ≥3
	Thrombocytopenia	32 (55%)	10 (17%)
	Lymphopenia	8 (14%)	7 (12%)
	Anemia	8 (14%)	0 (0%)
	Neutropenia	4 (7%)	2 (3%)
Nonhematologic AEs, n (%)	Nausea	32 (55%)	1 (2%)
	Diarrhea	29 (50%)	2 (3%)
	Asthenia	28 (48%)	3 (5%)
	Vomiting	26 (45%)	0 (0%)
	Decreased appetite	15 (26%)	2 (3%)
	Anemia	8 (14%)	0 (0%)
	Headache	8 (14%)	0 (0%)
	Constipation	7 (12%)	0 (0%)
	Dry mouth	7 (12%)	0 (0%)
	Hypokalemia	5 (9%)	4 (7%)
	Neutropenia	4 (7%)	2 (3%)
	Malignant neoplasm progression	3 (5%)	3 (5%)
	Gamma-glutamyltransferase increased	2 (3%)	2 (3%)

Six patients (10%) discontinued treatment due to AEs (4 due to thrombocytopenia, 1 due to vomiting and diarrhea, 1 due to cholangiolitis). No grade ≥3 prolongation of the QTc interval or grade ≥2 prolongation of the QTc interval persisting more than 14 days (as determined by a central electrocardiogram [ECG] reading center) was observed, and no patient discontinued due to QTc interval prolongation.

## DISCUSSION

Current treatments for patients with advanced metastatic solid tumors tend to be only palliative, which represents an area of unmet need. Concomitant chemoradiotherapy is an established treatment modality for solid tumors, particularly for loco-regional disease control [19]; however, the main drawback of chemotherapy when combined with radiotherapy is the possible amplification of radiation-induced acute and late toxicity to normal tissues. Thus, there is a need to optimize radiotherapy of metastatic solid tumors using combination agents that are active and tolerable.

Through preclinical studies in cancer models, HDACIs are emerging as radiation sensitizers, suggesting their clinical potential in combination with radiotherapy. Our preclinical data using an EMT-6 tumor-bearing murine model demonstrated that combination of low-dose radiation (2 Gy) and abexinostat led to inhibition of tumor growth and improved survival benefit compared with abexinostat alone or low-dose radiation alone (Supplementary Figures 1A and 1B). Previous studies demonstrated that following IV administration of radiolabeled [^14^C]-abexinostat, unchanged parent drug was the major component in the plasma, and radioactivity was widely distributed in all tissues, especially cerebellum, cerebrum, and cerebrospinal fluid, indicating that radiolabeled abexinostat crossed the blood-brain barrier (data not shown).

The current phase 1 study was designed to evaluate initial safety and tolerability of the novel HDACI abexinostat in combination with radiotherapy, with the intent of systemic disease assessment, such as secondary tumor sites in the setting of palliation, where long-term toxicities are different from those in early-stage localized disease [20]. The major limitation of the use of a trial design, such as ours, focusing on patients receiving radiation with a palliative intent (i.e., 30 Gy in 10 fractions of 3 Gy) without focusing on a specifically defined tumor location is that the trial generates information about normal tissue tolerance (the major objective of a radiation + new drug phase 1 trial), but only after irradiation of variable normal tissues and dose-limiting organs. Given the fact that the normal lung and bowel are major dose-limiting organs, but display different volume and radiation dose tolerance, the design of our trial included 2 distinct cohorts, the subdiaphragmatic group that is more likely to accrue patients for whom radiation fields encompass the bowel, and the supradiaphragmatic group that is more likely to include the lung into the irradiated volume [20]. However, we believe that this type of design was probably the safest in terms of risk/benefit balance when a drug is combined with radiation for the first time in humans. Previously used by other groups [21, 22], this type of design offers the advantage of a better safety balance when a drug is combined with radiation in human subjects for the first time. This is especially important because this type of trials select patients requiring palliative radiotherapy without concurrent chemotherapy (platinum, 5-fluorouracil, etc). On the contrary, a trial addressing solely one type of localized cancer treated with radiotherapy in a curative intent might have provided more information in terms of tolerance for a given organ; however, this type of trial would have inevitability carried the risk of major, permanent toxicity in a population of patients amenable to cure.

PK/pharmacodynamic modeling studies have been able to predict the optimal schedule of abexinostat allowing higher doses with minimal thrombocytopenia, showing that an abexinostat dosing schedule of 4 days on/3 days off (schedule 2 in the present study) was associated with smaller platelet decrease. The MTD2 reached for this optimized schedule was 105 mg/m^2^ BID, and the recommended dose, 90 mg/m^2^ BID [18]. These doses are in agreement with the findings of this study. We speculated that the unique PK profile of abexinostat (half-life 4-5 hours) with twice daily dosing [18] may provide an advantageous safety profile in combination with radiotherapy for the treatment of advanced solid tumors.

Safety data demonstrated good tolerability of abexinostat combined with hypofractionated palliative radiotherapy in this patient population of advanced solid tumors. No marked differences were observed in the frequencies of DLTs or AEs between the supra- and subdiaphragmatic groups. Thrombocytopenia was the most common hematologic toxicity, reported in 55% of the patients, with grade ≥3 in 17%. This result is consistent with the finding that thrombocytopenia is less frequently observed with the 4 days on/3 days off schedule [20] compared with a high frequency of grade ≥3 thrombocytopenia with a 14-day dosing schedule (21-day cycle) in patients with non-Hodgkin lymphoma and chronic lymphocytic leukemia [21]. The most common nonhematologic toxicities reported were nausea, diarrhea, asthenia, and vomiting, which have been commonly observed in previous studies with HDACIs. Cardiotoxicities such as QT/QTc prolongation have been a safety concern with the use of existing HDACIs in some instances [23, 24]. In the current study, no grade ≥3 prolongation of the QTc interval (as determined by a central ECG reading center) was observed. Only 2 patients on schedule 2 had a grade 1/2 prolongation of QTc, and no patient discontinued treatment due to QTc interval prolongation. QT prolongations (grade 2) in both patients resolved without dose reductions, were not serious, and were assessed by the investigator as unlikely related to the study treatment. Overall, only 10% of all treated patients discontinued treatment, demonstrating a tolerable safety profile of abexinostat in combination with radiotherapy.

The combination of abexinostat and low-dose radiotherapy led to a loco-regional response rate of 12%, with 5 PRs and 1 CR in the current study, and a substantial proportion of patients (63%) achieved a best loco-regional response of stable disease. A longer follow-up and a larger sample size is warranted to monitor these responses. No clinically significant differences in efficacy were observed between schedules 1 and 2 or supra/subdiaphragmatic groups. Due to the larger number of patients enrolled in some dose cohorts, a statistically valid conclusion could not be made for the dose/response relationship.

The ability of abexinostat to cross the blood-brain barrier demonstrated in pre-clinical studies is supported by the encouraging loco-regional responses observed in patients with brain lesions in this study and suggests that abexinostat in combination with radiotherapy may constitute a treatment option for these lesions. These findings are notable (particularly a best response of CR in 1 patient with brain lesions as non-target lesion) given the modest activity of currently approved HDACIs, vorinostat and romidepsin, as single agents or in combination therapy in patients with high-grade gliomas [25] and with glioblastoma [26–28].

Of note, HDACIs have been demonstrated to exert immunomodulatory effects in cancer cells, such as enhanced expression of natural killer cell–activating ligands, MHC class I and II molecules, and CD40 molecules [29–31]. It was demonstrated that vorinostat elicits antitumor immunity in colon cancer mouse models through immunogenic cell death [32]. The abscopal effect of radiation has been shown to be immune-mediated [33], in part by IL-12 [34], dendritic cells, and T cells [35]. Thus, the immunomodulatory effects of abexinostat have marked implications for radiotherapy and warrant evaluation of the combination in future radiotherapy studies. Subsequent trials with a focus on a particular tumor type are needed that can first test the combination in a phase I/II setting, especially if higher radiation doses or concurrent chemotherapy are investigated in combination with abexinostat.

In summary, oral abexinostat in combination with hypofractionated radiotherapy demonstrates activity with specific loco-regional responses in patients with metastatic solid tumors who have undergone prior surgery with chemotherapy or chemoradiotherapy. The combination is well tolerated, with the majority of AEs being grade 1/2. Although our patient population was small, based on the positive results observed, the potential of this combination for the treatment of patients with brain lesions should be investigated further in a larger population and with longer follow-up.

## MATERIALS AND METHODS

### Ethics statement

Investigation has been conducted in accordance with the ethical standards and according to the Declaration of Helsinki and according to national and international guidelines and has been approved by the authors’ institutional review board. Written consent was obtained from each patient prior to study entry.

### EMT-6 murine breast carcinoma model

Tumor volumes before and after treatment was assessed in 4 groups: vehicle only, abexinostat only, low-radiation only, and combination of low-dose radiotherapy and abexinostat (n=10 each). Female BALB/c mice 8 to 12 weeks old were injected in the flank region with 5 × 10^6^ EMT-6 tumor cells in 0% Matrigel (cell injection volume was 0.1 mL/mouse). Treatment was started on matched pairs when tumors reached an average size of 50-70 mm^3^. Tumors were irradiated at a dose of 2 Gy/animal for 5 days (1 cycle). Abexinostat (IP) was administered concurrently at a dose of 12 mg/kg BID, 6 hours apart, 5 days on/2 days off/week for 4 weeks. Mice were monitored until tumor volume reached 2000 mm^3^ or 45 days, whichever came first. Tumor volumes were measured twice per week in two dimensions using a calipers, and the volume was expressed in mm^3^ using the formula: V = 0.5 *a* × *b*^2^ where *a* and *b* are the long and short diameters of the tumor, respectively. Kaplan-Meier survival curves of EMT-6 tumor-bearing mice were analyzed until 45 days after the start of treatment.

### Study design

We conducted a multicenter, open-label, exploratory phase 1, dose-escalation study at 6 centers in Italy and France. Eligible patients had histologically confirmed solid tumors with measurable disease and required a course of hypofractionated palliative radiotherapy. These included patients with metastatic tumors with locally advanced disease for which primary tumor management before systemic treatment was required; primary tumors in relapse, tumors not previously irradiated, and tumors requiring hypofractionated radiotherapy; metastatic localization requiring hypofractionated radiotherapy; and patients not fit for conventional chemoradiotherapy. Other eligibility criteria included an Eastern Cooperative Oncology Group performance status ≤2; an estimated life expectancy >20 weeks; and adequate hematologic, cardiac, renal, and hepatic functions. Exclusion criteria included allogenic bone marrow transplant; major surgery within 4 weeks before first day of treatment; chemotherapy within 3 weeks (6 weeks in case of nitrosoureas) of study treatment administration; previous radiotherapy on the same area; immunotherapy or hormone therapy within 2 weeks except stable luteinizing hormone-releasing hormone agonist therapy for prostate cancer, stable oral glucocorticoid and mineralocorticoid replacement for adrenal insufficiency, stable mitotane for adrenal carcinoma, or oral contraceptives treatment with other anti-neoplastic agents within 3 weeks; concurrent therapeutic anticoagulation by vitamin K antagonists; any treatment known to prolong QTc interval; treatment with valproic acid within 5 days of study drug administration; and prophylactic use of growth factors.

### Abexinostat and radiotherapy schedule

Abexinostat in combination with a fixed radiation dose (30 Gy in 10 daily fractions of 3 Gy, 5 days per week over 2 weeks) was administered in 2 schedules. For schedule 1, treatment with abexinostat started 1 week before starting hypofractionated radiotherapy in order to induce biological modifications in tumor cells before radiation and then continued concomitantly with hypofractionated radiotherapy for 2 weeks. Abexinostat was given twice a day (BID) from day 1 to 5 and during radiotherapy from day 8 to 12 and 15 to 19. The schedule of 5 days on/2 days off (schedule 1) chosen for this study was based on tolerability and activity of abexinostat observed in the clinical study PCYC-0402 [18]. The choice of this schedule also allowed the administration of abexinostat concomitantly with radiotherapy. In order to increase safety margin of the combination of abexinostat with radiotherapy, we chose a starting daily dose of 15 mg/m^2^ BID for the present study, which is 25% of the recommended dose of 60 mg/m^2^ BID found in PCYC-0402 study and 1 dose level below the starting dose used in the PCYC-0402 study.

**Figure 1:**
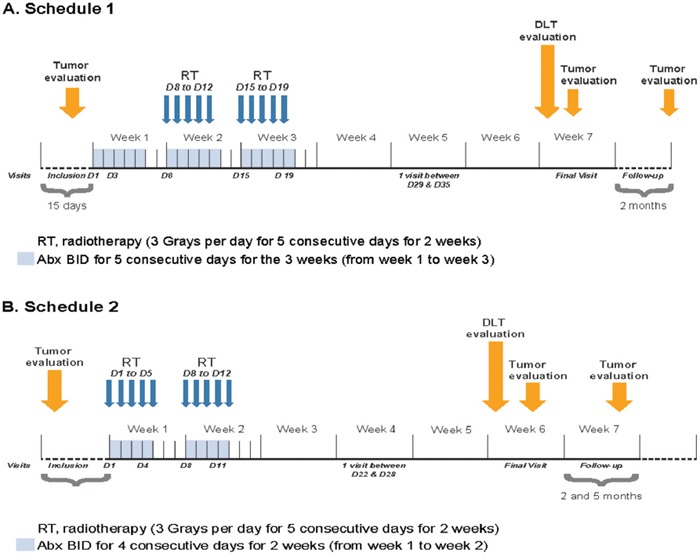
Study Design **A.** Schedule 1. **B.** Schedule 2.

For schedule 2, abexinostat and hypofractionated radiotherapy were administered concomitantly from day 1 to day 4 and from day 8 to day 11 (Figure 1). The schedule of 4 days on/3 days off (schedule 2) chosen for this study was based on tolerability of abexinostat observed in the clinical study CL1-78454-002 [20]. This schedule is associated with the smallest platelet decrease [20], and dose levels 75 mg/m^2^ BID, 90 mg/m^2^ BID, and 105 mg/m^2^ BID were investigated.

For both schedules, radiotherapy was administered using a 3-dimensional conformal technique. Patients received hypofractionated external beam irradiation using linear accelerators with photon energies ≥4 megavolts. Patients within the 2 schedules were categorized according to the localization of the radiation field (supradiaphragmatic and subdiaphragmatic group) in order to evaluate radiotherapy-related potential toxicities specific to the irradiated regions.

Dose escalation followed a 3 + 3 algorithm-based design and continued until maximum tolerated dose (MTD) was achieved based on protocol-defined dose-limiting toxicities (DLTs) defined as the occurrence in cycle 1 of any of the following: a grade 3 nonhematologic adverse event (AE), grade 3 prolongation of the QTc interval, grade 4 neutropenia lasting >5 days on growth factors, grade 4 thrombocytopenia, or failure to restart abexinostat administration within 2 weeks. Dose escalation to the next level proceeded after DLT assessment over the 3-week treatment period for schedule 1 or over the 2-week treatment period for schedule 2, and the following 3 weeks without treatment. Tumor evaluation was performed 3 weeks after treatment completion and after a 2-month follow-up. AEs at each visit were graded according to the Common Toxicity Criteria for Adverse Events [CTCAE] v3.0) [36]. Serious AEs (SAEs) were those events that were fatal, life threatening, required hospitalization, disabling, or judged to be medically significant.

### Study objectives and assessments

The primary objectives of the study were to assess the safety and tolerability of oral abexinostat given in combination with standard hypofractionated radiotherapy in patients with advanced solid tumor in terms of MTD and to establish the recommended phase 2 dose. The secondary objectives were to assess safety and tolerability of the combination, to evaluate tumor responses of the combination, and to analyze the pharmacokinetic (PK) profile of abexinostat and its metabolites. Tumor responses as defined by loco-regional response rate and overall response rate (ORR) were assessed per the revised Response Evaluation Criteria in Solid Tumors (RECIST version 1.1) [37].

ORR was defined as proportion of patients exhibiting a complete response (CR) or a partial response (PR) per the RECIST criteria (version 1.1). Loco-regional response rate was defined as the proportion of patients exhibiting a CR or a PR per the RECIST criteria (version 1.1) using tumor lesions encompassed within the radiation field. Lesions were evaluated by both clinical exam and computed tomography imaging. Electrocardiograms were performed at screening and both at pre-first dose and 1 to 1.5 hours post-first dose on days 1, 3, 8, 15, 19, and final visit of schedule 1 and days 1, 4, 8, 11, and final visit of schedule 2.

### Statistical analysis

The efficacy analyses were performed in the full analysis set, which included patients who had received at least 1 dose of study treatment and who had at least 1 baseline and 1 post-baseline tumor evaluation. Best overall response per investigator assessment was provided for each dose level and overall population by schedule and group. Response rates (loco-regional response and ORR) and corresponding exact binomial 95% confidence interval were provided. Safety analyses were conducted on the safety set in each anatomically based group by dose level. Safety was assessed with a description of DLTs on the DLT-evaluable population comprising patients who completed the total treatment period of 3 weeks for schedule 1 and 2 weeks for schedule 2 and 3 weeks without treatment period (i.e., evaluation period) following total treatment period or who discontinued due to DLT during total treatment or 3 weeks without treatment period. Descriptive statistics were used for evaluating toxicity (according to CTCAE v3.0), AEs, physical examination, performance status, vital signs, and laboratory tests.

## SUPPLEMENTARY TABLES AND FIGURES


