# Supplementary material for: A phase 1 dose-escalation study of the oral histone deacetylase inhibitor abexinostat in combination with standard hypofractionated radiotherapy in advanced solid tumors
Source: Oncotarget. 2016 Dec 24;8(34):56199–209. doi: 10.18632/oncotarget.14147 (PMC5593555; doi:10.18632/oncotarget.14147)
Supplement: Supplementary file 1 [file oncotarget-08-56199-s001.pdf]

A phase 1 dose-escalation study of the oral histone deacetylase inhibitor abexinostat in combination with standard hypofractionated radiotherapy in advanced solid tumors

SUPPLEMENTARY TABLES AND FIGURE

Supplementary Table 1: Number of patients enrolled by schedule and radiotherapy region

**Subdiaphragmatic (n=25)**					
	**Dose Level (BID)**	**# of Patients**		**Dose Level (BID)**	**# of Patients**
**Schedule 1**	15 mg/m^2	3	**Schedule 2**	60 mg/m^2	0
	30 mg/m^2	3		75 mg/m^2=120 mg	3
	45 mg/m^2	3		90 mg/m^2=140 mg	3
	60 mg/m^2	5		105 mg/m^2=160 mg	5
**Supradiaphragmatic (n=33)**					
**Schedule 1**	15 mg/m^2	3	**Schedule 2**	60 mg/m^2	6
	30 mg/m^2	4		75 mg/m^2 =120 mg	4
	45 mg/m^2	3		90 mg/m^2 =140 mg	3
	60 mg/m^2	9		105 mg/m^2=160 mg	1

Supplementary Table 2: Best loco-regional response and overall response by radiotherapy region

Best Loco-regional Response, n (%)	Supradiaphragmatic (n=29)	Subdiaphragmatic (n=22)
CR	0 (0.0%)	1 (5%)
PR	4 (14%)	1 (5%)
Stable disease (SD)	18 (62%)	14 (64%)
PD	5 (17%)	3 (14%)
Non CR/Non PD	0 (0%)	2 (9%)
Objective response rate	4 (14%)	2 (9%)
95% CI	3.9-31.7	1.1-29.2
**Best Overall Response, n (%)**		
CR	0 (0%)	1 (5%)
PR	3 (10%)	0 (0%)
SD	15 (52%)	12 (55%)
PD	11 (38%)	7 (32%)
Non-CR/Non-PD	0 (0%)	2 (9%)
Objective response rate	3 (10%)	1 (5%)
95% CI	2.2-27.4	0.1-22.8

Supplementary Table 3: DLTs by radiotherapy region and dose levels

Schedule 1 (n=27)							
		Subdiaphragmatic		Supradiaphragmatic		Total Numbers	
5d/7, 3w, 1 cycle Hydrochloride formula	Dose Level	# of Patients	# of DLTs	# of Patients	# of DLTs	# of Evaluable Patients	# of DLTs
	15 mg/m^2	3	0	3	0	6	0
	30 mg/m^2	3	0	4 (1 NE)	0	6	0
	45 mg/m^2	3	0	3	0	6	0
	60 mg/m^2	5 (2 NE)	0	9 (3 NE)[b]	2	9	2
**Schedule 2 (n=23)**							
		**Subdiaphragmatic**		**Supradiaphragmatic**		**Total Numbers**	
4d/7, 2w, 1 cycle Hydrochloride formula/ tosylate formula	**Dose Level**	**# of Patients**	**# of DLTs**	**# of Patients**	**# of DLTs**	**# of Evaluable Patients**	**# of DLTs**
	60 mg/m^2	-	-	7 (1 NE)	1	6	1
	75 mg/m^2 =120 mg[a]	3	0	5 (2 NE)	0	6	0
	90 mg/m^2 =140 mg[a]	3	0	4 (1 NE)	0	6	0
	105 mg/m^2 =160 mg[a]	5 (1 NE)[c]	2	1	1	5	3

DLTs, dose-limiting toxicities; NE, not evaluable.
[a]Equivalence in tosylate formula.
[b]MTD1 for schedule 1 was reached at 60 mg/m^2 in supradiaphragmatic group.
[c]MTD2 of schedule 2 was reached at 105 mg/m^2 (160 mg[a]) for subdiaphragmatic group.

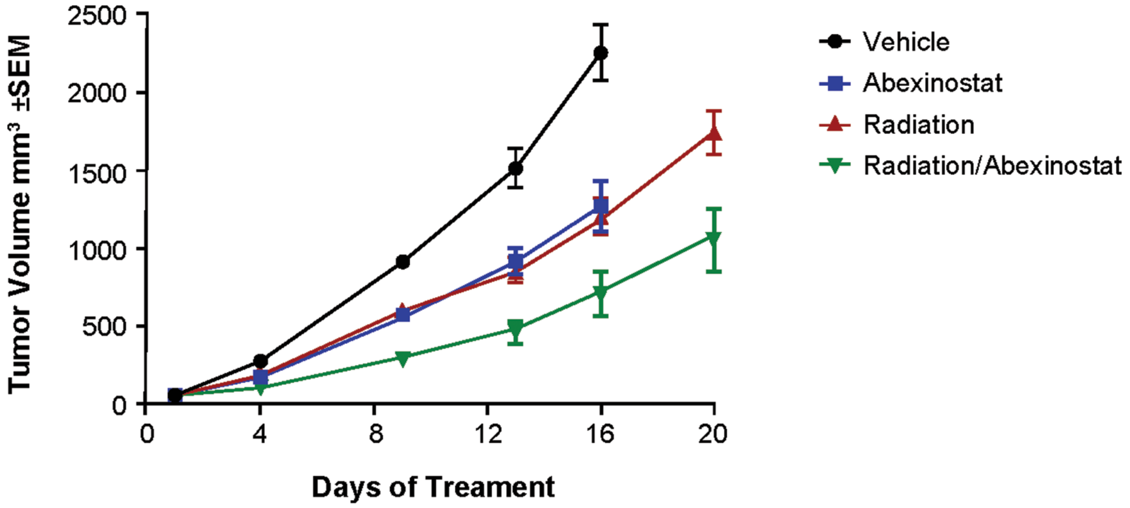

Supplementary Figure 1A: Mean tumor growth inhibition in murine breast cancer model (EMT-6).

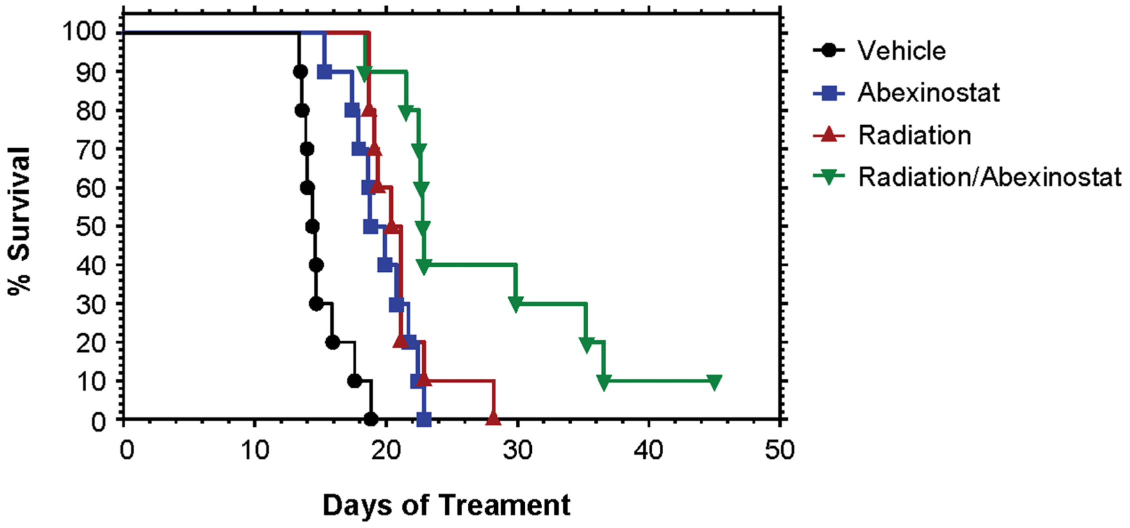

Supplementary Figure 1B: Kaplan-Meier survival curve in murine breast cancer model.
